# Coordination Aspects of an Effective Sprint Start

**DOI:** 10.3389/fphys.2018.01138

**Published:** 2018-08-17

**Authors:** Zbigniew Borysiuk, Zbigniew Waśkiewicz, Katarzyna Piechota, Paweł Pakosz, Mariusz Konieczny, Monika Błaszczyszyn, Pantelis T. Nikolaidis, Thomas Rosemann, Beat Knechtle

**Affiliations:** ^1^Faculty of Physical Education and Physiotherapy, Opole University of Technology, Opole, Poland; ^2^The Jerzy Kukuczka Academy of Physical Education in Katowice, Katowice, Poland; ^3^Exercise Physiology Laboratory, Nikaia, Greece; ^4^Institute of Primary Care, University of Zurich, Zurich, Switzerland; ^5^Medbase St. Gallen Am Vadianplatz, St. Gallen, Switzerland

**Keywords:** EMG, reaction time, auditory stimuli, movement patterns, level of performance

## Abstract

The aim of the present study was to examine differences in the movement structure and the coordination aspects expressed by bioelectrical tension indicators between a group of experts/sprinters and a group of novices/students. A group consisted of 20 sprinters and a control group consisted of 35 master’s physical education students. A 16-channel surface electromyography (14 muscles) and two cameras with recording speed of 250 frames/per second were used. Significant differences were found between reaction time (*p* < 0.005) and time at 30 m of the covered sprinting distance (*p* < 0.001) between the students and advance athletes. Furthermore, the sprinters activated the back foot (taking off the starting blocks) and the front foot (first ground contact) earlier, which correlated with the attained times at 5 (*r* = 0.66) and 10 m (*r* = 0.62) of the covered sprinting distance. The most important component of the sprint start, apart from the muscle strength of the legs, is the appropriate motor coordination, which greatly affects the generation of power in the legs at the right time and optimal duration.

## Introduction

Sports professional experiences are leading to adaptive changes in the human central nervous system (CNS) and on the autonomic nervous system. The changes of the cortical excitability are significantly influence to differences between athletes and novices (Sessa et al., 2018).

Sprinting training, apart from developing physical performance, also involves perfecting all speed components – reaction time, movement speed and movement frequency – as determinants of the temporal structure of the sprint start (Collet, 1999). An effective sprint start requires a quick reaction to an auditory stimulus followed by a determined sequence of activation of particular muscles responsible for maximum movement speed.

Surface electromyography (sEMG) can be used for accurate measurements of sprinters’ start reaction to auditory stimuli. Reaction time (RT) in EMG is regarded as an interval between the appearance of the stimulus and the first bioelectrical activity of the muscle. The detailed analysis of the initial phase of a sprint run was carried out. The sprint start was regarded as the time from the commands “On your marks” and “Get set” until the start signal and taking off the starting blocks. According to many authors, the start reaction time significantly affects the reduction of running time in 100 m races (Pilianidis et al., 2012; Tonnessen et al., 2013).

On the contrary, Maćkała and Mero (2013) suggested that sprint start effectiveness depends on the performance and the strength of the involved muscles of the arms and the legs. They point to the impact of technical skills on triggering off speed and strength abilities in athletes. They showed that the start acceleration (64%) and maintaining of peak speed (18%) were most significantly associated with the final results of a 100 m run. On the other hand, the least correlated with the 100 m final results were taking off the starting blocks (5%) and reaction time (1%). A further study (Maćkała, 2007) confirmed that speed endurance had a more significant effect on running time than speed and starting skills, i.e., technical skills.

Similar studies were also conducted to investigate determinants of the sprint start, focusing mostly on start acceleration, running the distance, and breaking the finish line. These authors also examined the reaction to auditory stimulation and taking off the starting blocks as a manifestation of the kinematic force of the legs. A number of authors investigated the start technique in short-distance runners (Korchemny, 1992; Reis and Fazenda, 2004). They reported on the distribution and volume of the ground forces during crouch starts (Korchemny, 1992) and on correlations between the first strides during a run and acceleration variables.

Some researchers claim that shorter reaction times lead to a shorter running time in short-distance races (Collet, 1999). Auditory reaction time (acoustic perception) determines the speed of movement performance among novice sprinters (120–160 ms), and among elite sprinters (50–100 ms). According to Čoh and Tomažin (2006) an elite 60 m runner (during indoor world championships) reached a reaction time of 124 ms. Depending on the levels of reactions to different stimuli (auditory, visual, sensory) the time of stimulus reception by the brain is also related to the distance covered by the emitted stimulus (Kosiński, 2006). It was proven that simple reaction time can take place within an interval of 85 ms, and EMG latency can be even shorter up to 60 ms (Pain and Hibbs, 2007).

Key parameters which directly determine the time of a sprint run not only include the mentioned start reaction time but also the bioelectrical activity of muscles (EMG signal) and their timing. The reading of the EMG signal synchronized with speed cameras allows a multiple verification of movement sequences on the basis of their technical performance patterns.

Using a novice-expert paradigm, the present study investigated a group of physical education college students after two semesters of track and field classes as well as elite sprinters at a specialist stage of their training. It was assumed that start reaction time and timing of activation of selected muscles have a significant effect on sprinting performance.

## Materials and Methods

### Ethical Approval

The study was approved by the Bioethical Committee of the Physicians’ Chamber in Opole, Poland (Decision no. 208, June 5, 2014). The subjects gave informed consent to the study.

### Participants

Fifty five college students took part in the study: a group of 20 *advanced male* sprinters (1st sports class) and a control group of 35 master’s physical education male students. Group 1 consisted of advanced sprinters: The sprinters’ mean age: 22.50 (*SD* ± 2.56), body mass: 80.00 kg (*SD* ± 8.97), body height: 182.65 cm (*SD* ± 7.64), and mean BMI: 23.91 kg/m^2^ (*SD* ± 1.52). The length of sprinters’ training experience amounted to: 6.63 (*SD* ± 2.98). Group 2 served as controls – graduate physical education students: The students’ mean age: 24.43 (*SD* ± 0.64), body mass: 78.66 kg (*SD* ± 9.64), body height: 182.97 cm (*SD* ± 6.70), and mean BMI: 23.47 (*SD* ± 2.34). None of the students were high-performance athletes.

### Procedures

The testing apparatus consisted of a 16-channel surface electromyography (NORAXON, MyoTrace 400). In the present study only 14 surface electrodes were used, 7 on each side of the body. The EMG with a signal registering unit was coupled with two cameras with a recording speed of 250 frames per second (Point Grey Gazell).

The EMG and the cameras were connected directly with the CRI EVA computer (*Computer 1*) with a CRI VIST synchronizer. The starting signals to which the subjects responded were configured using an extra sound recorder (for Mics software) and a microphone with a computer (*Computer 2*) which emitted the sound. The total video analysis was conducted with the use of ProAnalyst software.

The electrodes were placed on a given muscle in accordance with SENIAM standards. For setting the baseline (Onset/Offset) of muscle activation, the MyoResearchsoftware (XP Mater Edition) was used. The Onset and Offset values were determined on the basis of local peak value – 5% (De Luca et al., 2010).

### Methods

The use of EMG allowed examination of performance of the following muscles of the right and left body side: (1) flexors and extensors of the arms: m. biceps brachii (BB) and m. triceps brachii (TB) and (2) flexors and extensors of the legs: m. rectus femoris (RF), m. vastus lateralis (VL), m. biceps femoris (BF), m. semitendinosus (STN), and m. gastrocnemius medialis (GAS MED).

Each main session of the study was preceded by a 20-min general warm up consisting of stretching and fitness exercises for the head, arms, trunk, legs, hips, and knees. The surface electrodes and EMG sensors were placed on participants’ skin. The participants then performed three sprinting trials over a distance of 30 m, with 2 to 4-min rest breaks, depending on one’s individual HR.

The start reaction times were determined on the basis of video recordings from two cameras (first recorded visible movement activated by arm muscles, lifting the back foot and the front foot from the starting blocks, the contact of both feet with the ground as time necessary to cover the first two strides after the start). Finish times were recorded at 5, 10, and 30 m after the start. Using the EMG system, the timing of bioelectrical activity of selected muscle groups was recorded. The EMG signal of the muscles involved in the sprint start were noted down as single continuous recordings from taking off the starting blocks to the completion of the first two strides. Each participant’s best trial result recorded by the EMG system and cameras was taken for detailed analysis.

### Statistical Analysis

The normality of distribution of variables was checked with the Shapiro–Wilk test. Since not all variables conformed to normal distribution, the non-parametric Spearman’s rank correlation coefficient (R) was used. If a pair of variables did not conform to normal distribution, the *U* Mann–Whitney test was applied.

## Results

**FIGURE 1:**
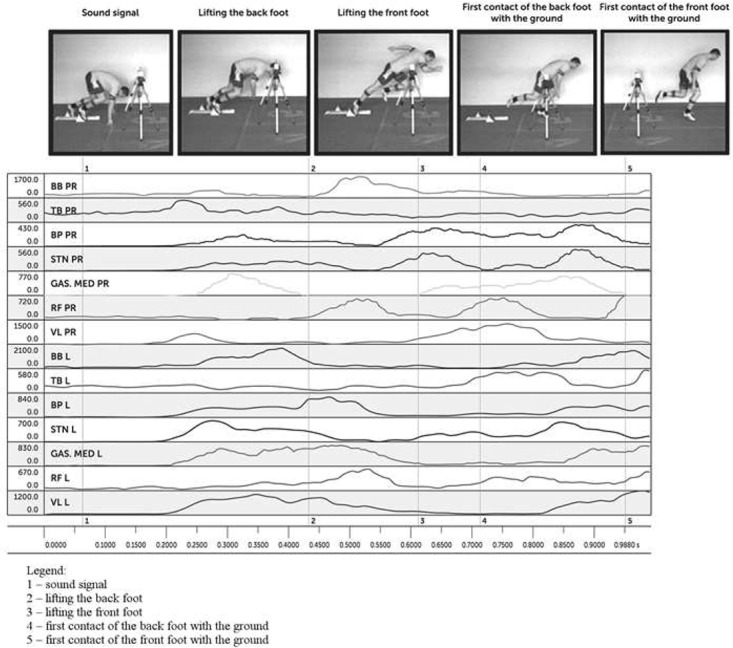
Movement pattern of the sprint start of the best elite runner. Muscle abbreviations: BB, m. biceps brachii;, TB, m. triceps brachii; RF, m. rectus femoris; VL, m. vastus lateralis; BF, m. biceps femoris; STN, m. semitendinosus; GAS MED, m. gastrocnemius medialis; L, left side; PR, right side. The subject gave written informed consent for the publication of the image.

**Figure 1** shows a sequence of movements by the best elite sprinter in the study. The data was recorded by a video-EMG integrated system. Each video frame (1, 2, 3, 4, and 5) corresponded to the time of a given sequence during the whole movement from the command “Get set” until the first two strides after the start. The first sequence was analyzed in detail: from the start signal (1st time – 0.061 s), lifting the back foot (2nd time – 0.37 s), lifting the front foot (3rd time – 0.61 s), first contact of the back foot with the ground (4th time – 0.71), and first contact of the front foot with the ground (5th time – 0.98 s).

**Table 1** shows the variables affecting sprint start timing among sprinters and students. Reaction time was a direct determinant of the final running result. Moreover, the time recorded at 30 m was shown to significantly affect acceleration and starting speed in sprint runs.

**Table 1:** Statistics of timing variables of sprinter’s and student’s start.

*SD* variable	Descriptive statistics (Sprinters)					Descriptive statistics (Students)				
	*N*	Mean	*SD*	Min	Max	*N*	Mean	*SD*	Min	Max
Start reaction time [s]	20	0.152	0.021	0.131	0.197	35	0.169	0.027	0.131	0.247
Lifting the back foot [s]	20	0.357	0.046	0.272	0.460	35	0.400	0.046	0.316	0.520
Lifting the front foot [s]	20	0.542	0.054	0.451	0.697	35	0.610	0.049	0.539	0.736
First ground contact of the back foot [s]	20	0.617	0.051	0.545	0.754	35	0.664	0.050	0.589	0.781
First contact of the front foot with the ground [s]	20	0.875	0.060	0.782	1.003	35	0.932	0.067	0.824	1.107
Time at 5 m [s]	20	1.65	0.09	1.50	1.91	35	2.36	0.42	1.19	3.11
Time at 10 m [s]	20	2.33	0.11	2.16	2.64	35	3.08	0.44	1.91	3.86
Time at 30 m [s]	20	4.18	0.16	3.85	4.65	35	4.35	0.21	4.03	5.05

The elite sprinters being studied attained the start reaction time of 0.152 ± 0.021 s. The sprint start timing was considered a coordinated sequence of movements from lifting the back foot (0.357 ± 0.046 s) to the first contact of the front foot with the ground (0.875 ± 0.060 s). Thus the time of the entire sequence was 0.518 s (**Table 1**).

The students’ mean start reaction time was 0.169 ± 0.027, lifting the back foot 0.400 ± 0.046 s, and the time of completing the start phase by the first contact of the front foot with the ground was 0.932 ± 0.067 s. The students began the visible phase of the sprint start significantly later (at 0.043 s), and also completed the phase significantly later (at 0.057 s) than the sprinters (**Table 1**).

The time of start reaction to an auditory stimulus was significantly different (0.017 s; Mann–Whitney *U* test, *Z* = −3.185, *p* = 0.001) between the sprinters and controls (students). The time at 30 m for the sprinters amounted to 4.18 ± 0.16 s (Mann–Whitney *U* test, *Z* = −2.783, *p* = 0.005) whereas the students’ time was significantly longer with 4.35 ± 0.21 s. The time difference between the two study groups was 0.17 s. Differences were also noted between the groups at 5 and 10 m of the covered distance (**Table 1**).

Strong and moderate correlations were found for the group of sprinters between start reaction time and activity of leg muscles in the starting phase (*R* = 0.77, 0.67, 0.53, 0.41) (**Table 2**). No significant correlations were noted between start reaction time and times at 5, 10, and 30 m of the covered distance. However, significant correlations can be noted between the bioelectrical activity of the legs and the times at 5 and 10 m.

**Table 2:** Spearman’s rank correlations between start time variables in advanced sprinters.

Variable	Spearman’s rank correlation (Sprinters)							
	Start reaction time [s]	Lifting the back foot [s]	Lifting the front foot [s]	First ground contact of the back foot [s]	First ground contact of the front foot [s]	Time at 5 m [s]	Time at 10 m [s]	Time at 30 m [s]
Start reaction time [s]	1.00	0.77	0.67	0.53	0.41	0.32	0.28	0.15
Lifting the back foot [s]	0.77	1.00	0.87	0.81	0.76	0.54	0.52	0.11
Lifting the front foot [s]	0.67	0.87	1.00	0.91	0.88	0.67	0.69	0.23
First ground contact of the back foot [s]	0.53	0.81	0.91	1.00	0.82	0.61	0.66	0.13
First ground contact of the front foot [s]	0.41	0.76	0.88	0.82	1.00	0.60	0.62	0.17
Time at 5 m [s]	0.32	0.54	0.67	0.61	0.60	1.00	0.98	0.40
Time at 10 m [s]	0.28	0.52	0.69	0.66	0.62	0.98	1.00	0.42
Time at 30 m [s]	0.15	0.11	0.23	0.13	0.17	0.40	0.42	1.00

Moderate correlations were found for the group of students between start reaction time and activity of leg muscles in the starting phase *R* = 0.51, 0.37, – 0.41, 0.36; and weak correlations between start reaction time and times at 5 and 10 m of the covered distance (**Table 3**). Like for the sprinters, strong correlations were noted between determinants of the timing of leg muscle activity.

**Table 3:** Spearman’s rank correlations between start time variables physical education students (controls).

Variable	Spearman’s rank correlation (Students)							
	Start reaction time [s]	Lifting the back foot [s]	Lifting the front foot [s]	First ground contact of the back foot [s]	First ground contact of the front foot [s]	Time at 5 m [s]	Time at 10 m [s]	Time at 30 m [s]
Start reaction time [s]	1.00	0.51	0.37	0.41	0.36	−0.36	−0.35	−0.07
Lifting the back foot [s]	0.51	1.00	0.88	0.91	0.87	0.11	0.14	0.32
Lifting the front foot [s]	0.37	0.88	1.00	0.88	0.94	0.26	0.29	0.31
First ground contact of the back foot [s]	0.41	0.91	0.88	1.00	0.91	0.17	0.20	0.31
First ground contact of the front foot [s]	0.36	0.87	0.94	0.91	1.00	0.24	0.27	0.27
Time at 5 m [s]	−0.36	0.11	0.26	0.17	0.24	1.00	1.00	0.44
Time at 10 m [s]	−0.35	0.14	0.29	0.20	0.27	1.00	1.00	0.45
Time at 30 m [s]	−0.07	0.32	0.31	0.31	0.27	0.44	0.45	1.00

Most importantly, there was no correlation in the control group (students) between the bioelectric muscle activity of the legs during the sprint start and the times at 5 and 10 m of the covered sprinting distance. It might be assumed that a higher level of neuromuscular coordination displayed by the sprinters determined a greater effectiveness of running performance at 5 and 10 m of the covered distance.

## Discussion

Sprint start movement patterns indicate that the bioelectrical activities of biceps femoris and semitendinosus muscles are significantly correlated during a post-start phase, from lifting the front foot to the completion of the first two strides, or even at 5 and 10 m of the covered distance. Sciatic-tibial muscles are responsible for knee flexion, and thus for prolonging the midflight phase of the back foot during a sprint run. In turn, the gastrocnemius medialis muscles display similar correlations after the start phase. They become activated in the support phase, and remain active during the run until the next stance. They are usually activated while exerting pressure with the feet on the starting blocks, and are mostly responsible for ankle rotation as well as knee flexion and blocking (Wiemann and Tidow, 1995). On the other hand, the vastus lateralis is activated during a quick start reaction, and the rectus femoris at 10m of the running distance. These two muscle groups control extension of the leg, and actively participate in movement between the commands “On your marks” and “Get set.” The whole center of gravity becomes “lifted” and “shifted” to the arms. This leads to an activation of biceps and triceps brachii, rectus femoris, vastus lateralis, and gastrocnemius medialis muscles (Mero and Komi, 1990; Francavilla et al., 2018).

The motor profile of sprinters and physical education students discussed above must be complemented with technical preparation attributes referred to in the present study as sprint start timing. The sequence of activation of particular arm and leg muscles is an explicit manifestation of the level of neuromuscular predispositions and of recognition of the right intervals during motor pattern timing (Magill, 1993; Monda et al., 2017). Sprinting is a motor skill which can be learnt, whose timing is closely associated with movement precision and accuracy. According to Rimmer and Sleivert (2000) coordination preparation develops the ability to combine and harmoniously coordinate motor activities, and it enhances the maximum utilization of sprinter’s potential. All distortions and reductions in the coordination of a single movement in the sprinting stride cycle result in the delaying of the start, as well as stance and swing phases (Sessa et al., 2018).

Movement coordination is a crucial component associated with full psychophysical readiness and attention concentration (Starkes and Ericsson, 2003). Also, the programming of the training process, especially at the specialist stage, is highly significant (Husbands, 2013). Training must account for motor coordination and psychophysical concentration. The interaction between the runner and the starting blocks greatly influences the sprint start effectiveness. The time interval between the commands “Get set” when the runner is motionless until the starting signal is usually from 1 to 2 s. During this short time the runner should attain the state of full concentration and psychomotor readiness. Ozolin (1988) claimed that the runner need 0.22–0.45 s between hearing the signal and leaving the starting blocks. This time depends primarily on the sprinter’s speed and smoothness of coordination of the arms and the legs, as well as the psychophysical predispositions. The mastery of sprint start motor habits also significantly affects sprint start effectiveness (Ward and Radford, 2000; Jeffreys, 2013).

The present study also involved an analysis of variables affecting sprint start timing among sprinters and students. Reaction time is a direct determinant of the final result of the run; moreover, the time at 30 m of the covered running distance has a significant effect on acceleration and starting speed in sprints. Henry (1952) observed that the most important component of the sprint start, apart from the muscle strength of the legs, is the appropriate motor coordination between lifting of the back foot and the front foot. This greatly affects the generation of power in the legs at the right time and with optimal duration.

Reaction time was defined by Brüggemann and Glad (1990) as the interval between the manifestation of the stimulus (starting signal) until the sending of the impulse to the muscles. In the present study, the muscles of the arms were activated first. Some authors claim that RT should be concluded with muscles exerting pressure on the starting block (Ditroilo and Kilding, 2004). Furthermore, according to Čoh et al. (2006) a sprinter’s total reaction time consists of the so-called *premotor time* – from the starting signal to the first activation of the muscles of the legs – and *motor time* involving intense muscle tension until the back foot and the front foot have left the starting blocks. Husbands (2013) state that reaction time is an interval between the manifestations of a stimulus until the start of the movement. Müller and Hommel (1997) indicate that a very good start reaction time for elite sprinters is below 0.140 s, while a poor start reaction time is above 0.190 s. Tellez and Doolittle (1984) estimate the contribution of reaction time to the final outcome of a sprint run at 2–3%.

Start reaction time is the natural commencement of the runner’s movement. It depends primarily on sprinters’ individual traits and neuromuscular predispositions (Maćkała and Mero, 2013). The shorter the reaction time, the better the sprinting time is. Previously, such authors as Mero et al. (1992) thought that a short reaction time, even in very good sprinters, only slightly influences their overall sprinting effectiveness. The main determinant in their opinion was the generation of maximal force and starting speed. Therefore, a crucial factor directly influencing sprinting performance is attainment of peak start acceleration within the first 25–30 m. The world’s elite sprinters reach 50–55% of maximal speed in the first 10 m, 70–80% between 10 and 20 m, and 85–95% by 30 m (Čoh and Tomažin, 2006; Pavlović et al., 2013).

The present study revealed moderate differences between the advanced sprinters and college students. The mean start reaction time in the sprinters’ group was 0.152 s, whereas in the students’ group it amounted to 0.169 s. Thus the inter-group difference was only 0.017 s. A detailed analysis of the study results showed that the shortest start reaction time in both groups was 0.131 s in accordance with the criteria provided by Susanka et al. (1989).

A start reaction time between 0.130 and 0.150 s is regarded as an above average auditory reaction. Reis and Fazenda (2004), who compared groups of athletes and students, admitted that simple reaction time is determined by athletes’ individual predispositions. Reis and Fazenda (2004) also think that start reaction time does not significantly affect (*R* = 0.22) the 100 m running performance of novice and medium-level athletes. In other words, sprint start effectiveness at the general and specialist training phases should be associated with the maximal use of athletes’ strength-speed and coordination skills, as confirmed by the results of the present study. There are significant relationships between the start reaction time and the quick, coordinated movements of the legs after taking off the starting blocks for the group of studied sprinters. This is an indication of the importance of starting technique for elite runners. A reverse trend was noted in the control group (college students).

It can be concluded that the performance determinants of novice sprinters could be significantly correlated with a short time at 30 m of the covered running distance. The obtained study results indicate that simple reaction time as an isolated factor has a limited impact on sprint start effectiveness.

## Author Contributions

ZB, ZW, and BK designed the study. ZB, KP, and PP collected and analyzed the data. ZB, ZW, KP, MK, MB, PN, TR, and BK interpreted the data and prepared the manuscript. All the authors approved the final version of the paper.

## Conflict of Interest Statement

The authors declare that the research was conducted in the absence of any commercial or financial relationships that could be construed as a potential conflict of interest.
